# On the complementarity of X-ray and NMR data

**DOI:** 10.1016/j.yjsbx.2020.100019

**Published:** 2020-01-07

**Authors:** Antonio Schirò, Azzurra Carlon, Giacomo Parigi, Garib Murshudov, Vito Calderone, Enrico Ravera, Claudio Luchinat

**Affiliations:** a Magnetic Resonance Center (CERM) and Interuniversity Consortium for Magnetic Resonance of Metallo Proteins (CIRMMP), Via L. Sacconi 6, 50019 Sesto Fiorentino, Italy; b Department of Chemistry “Ugo Schiff”, University of Florence, Via della Lastruccia 3, 50019 Sesto Fiorentino, Italy; c MRC Laboratory for Molecular Biology, Francis Crick Ave, Cambridge CB2 0QH, UK

**Keywords:** Structure refinement, Integrated structural biology, RDC, X-ray, REFMAC

## Abstract

X-ray crystallography and NMR contain complementary information for the structural characterization of biological macromolecules. X-ray diffraction is primarily sensitive to the overall shape of the molecule, whereas NMR is mostly sensitive to the atomic detail. Their combination can therefore provide a stronger justification for the resulting structure. For their combination we have recently proposed REFMAC-NMR, which relies on primary data from both techniques for joint refinement. This possibility raises the compelling question of how far the complementarity can be extended. In this paper, we describe an integrative approach to the refinement with NMR data of four X-ray structures of hen-egg-white lysozyme, solved at atomic resolution in four different crystal forms, and we demonstrate that the outcome critically depends on the crystal form itself, reflecting the sensitivity of NMR to fine details.

## Highlights

• X-ray diffraction and NMR spectroscopy are complementary sources of information.
• Even at atomic resolution, NMR data improves the accuracy of X-ray structures.
• Real local discrepancies between solution and crystals are highlighted.
• For HEWL, a better agreement is found for the crystal forms with looser packing.

## Graphical abstract

**Figure:**
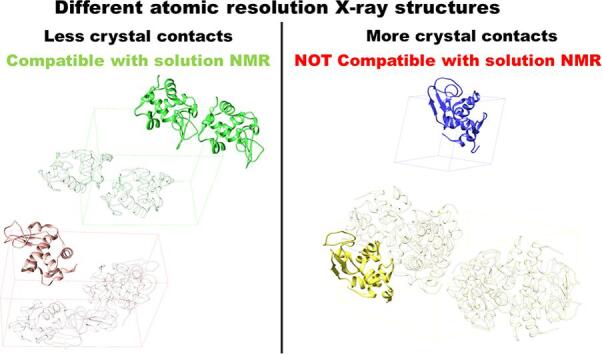

## 1 Introduction

X-ray crystallography and NMR are the two most common experimental sources for the structural determination of biological macromolecules, accounting for 89% and 8% of the PDB as of August 2019.^2^ These two structural techniques are not just alternative to one another, because they contain completely different information. The structural (electron density) information is encoded in X-ray data (reflections) through the Fourier transformation between direct and reciprocal spaces: in such a way the first/stronger reflections that are obtained at narrow diffraction angles (i.e.: low resolution) mostly encode information on the global level, and the reconstruction proceeds towards the electronic density of individual atoms as high quality reflections extend to wider and wider diffraction angles (see, for an introductory discussion, paragraph 2.4 in (Rhodes, 2006)). On the contrary, the structural information in NMR is mostly encoded as interatomic distances or bond orientations, and therefore NMR gains local information much before the global information can be reconstructed (see, for an introductory discussion, paragraph 10.2.1 in (Cavanagh et al., 2007)): the local information from NMR is substantially more precise than what it is possible to obtain from a crystallographic experiment, whereas the crystallographic experiment directly provides an overall image without having to build it up from a network of short distances. The other point of complementarity is a direct consequence of the above: X-ray diffraction aims at reconstructing the electron density in the crystal from the structure factors (scattering amplitudes) in the reciprocal space, and the structure factor increases with the square of the atomic number, therefore the response from hydrogen atoms is far smaller than that from heavier atoms (Fig. S1). The positions of hydrogen atoms could be obtained at ultrahigh resolution from Fo-Fc difference electron density maps. In practice, however, even in the highest resolution macromolecular X-ray structures, only a limited number of hydrogen atoms positions can be experimentally determined, only within well-ordered regions at the core of proteins (Howard et al., 2004). Otherwise, the positioning of hydrogen atoms must rely upon some prior information^3^; on the other hand, NMR data often start from hydrogens, because of the higher sensitivity of ^1^H spectroscopy, and their relative position with respect to heavier atoms can be obtained with high accuracy with minor effort (Zweckstetter and Bax, 2002), relying on prior knowledge about the location of the heavy atoms. In this work we consider the use of residual dipolar couplings (RDCs) measured on amide protons. RDCs arise as a result of partial molecular alignment, which can occur because of the interaction of the molecule of interest with a strongly oriented objects in solution or because of field-dependent alignment of the molecule itself. Each RDC reports on the alignment of the internuclear vector in the frame provided by the molecular alignment tensor (Bax, 2003). Amide RDCs thus report on the position of the H-atom relative to the backbone and therefore may improve the identification of H-bonded pairs, as well as providing hints on the preservation of the H-bonding pattern (vide infra). In this work we have used RDCs to refine only the direction of the NH bond, not its length: in the RDC calculation the NH bond length is kept fixed at 1.02 Å. X-ray data and NMR intrinsically encode non-overlapping information, even at the currently achievable highest resolutions.

Building on these considerations, several groups involved in the development of Integrative Structural Biology have worked towards a profitable integration of the two techniques (Brunger et al., 1987, Shaanan et al., 1992, Chou et al., 2000, Chou et al., 2001, Prestegard et al., 2005, Skrynnikov et al., 2000, Tian et al., 2001, Ulmer et al., 2003, Bertini et al., 2009). Among those we have included the possibility of using RDCs (as well as pseudocontact shifts for paramagnetic proteins) (Carlon et al., 2018, Kovalevskiy et al., 2018, Rinaldelli et al., 2014), in REFMAC (Murshudov et al., 2011, Murshudov et al., 1997), one of the most commonly used software for X-ray structure refinement.^4^

Through REFMAC-NMR we have found that, very often, X-ray structures and NMR data are compatible with one another within the experimental uncertainties of the two techniques (Carlon et al., 2018, Carlon et al., 2016, Rinaldelli et al., 2014). In such cases, the resulting refined structures become largely compatible with both datasets. In other cases, even upon joint refinement, the X-ray structure remains incompatible with the solution data and one can expect that, when different X-ray structures are available with differences in coordinates larger than their respective uncertainty, only a subset of those will be a proper representation of the molecule in solution. In turn, a reduction of the discrepancy between the two methodologies allows for highlighting those inconsistencies that cannot be reconciled (Zweckstetter and Bax, 2002) and that, therefore, are to be attributed to real structural differences between the crystal and the solution. In the REFMAC-NMR approach, the NMR data are fit to a single static model, and mobility effects are not included in the calculation. Molecular motions are expected in any physical system because of the intrinsic distribution of the thermal energy, therefore by the joint refinement we are not denying that mobility is present. However, we try to account for as much as possible of the data without invoking mobility, therefore reducing the risk of overinterpretation of the NMR data: according to a previous analysis, the NMR data used in the present work contain enough information to accurately identify the orientation of NH bonds but not to recover information about mobility (Higman et al., 2011).

Resolving the biases from each technique yields a structure that is more accurate than what could be obtained by either method and that, at the same time, preserves the information from both techniques (Mitchell and Gronenborn, 2017). A recent work on the philosophy of integrative techniques has stated the following about joint refinement: “the blind spots of x-ray crystallography and NMR cannot be removed, but when they are system relative, then they can be exposed by the mutual analysis of joint refinement […] At the end of joint refinement, there is not global unification of two methods into one or reduction or elimination of two methods to a single perspective. […] The information afforded by a phenomenon to the different modalities when jointly integrated can yield more accurate information than any one sense could ever yield” (Mitchell, 2019).

It is important to remark that, in REFMAC-NMR, hydrogens are positioned according to the NMR data, but they are bound to respect tight geometrical restraints, which are even tighter than those usually applied in the X-ray crystallography process. This implies that, when an amide hydrogen is moved from the library position, the backbone of the protein is moved to accommodate this change, of course within the boundaries imposed by the electron density. Since the electron density for lower resolution structures is broader and can accommodate larger changes, structures with lower resolution can be modified more easily, whereas we may expect that higher resolution structures will be more difficult to modify if the X-ray data and the NMR data are even slightly incompatible. This consideration poses the question of how far it is possible to modify an X-ray structure, and what is the meaning of the refined structure, especially when the structure itself is solved at atomic resolution. To answer this question we have selected the case of hen-egg white lysozyme, for which many high resolution structures have been deposited (783 PDB entries obtained by X-ray diffraction as of August 2019, of which 168 below 1.5 Å resolution and 13 at atomic resolution), and for which 8 datasets of RDCs are available (Schwalbe et al., 2001). The result of the joint refinement is evaluated in terms of the agreement with both experimental sources, and a refinement is considered satisfactory if a good agreement is obtained for NMR without altering significantly the agreement to X-ray. The results were evaluated globally, to determine initially which structures yield a satisfactory joint refinement and which did not (Rinaldelli et al., 2014). A good global agreement of a single model with both the NMR observables and the X-ray data, ensured that most of the NMR data were satisfied. This allowed for those that are violated to stand out, hence allowing the results to be analyzed in terms of the local effects (Carlon et al., 2016).

## 2 Materials and methods

The structure and the corresponding structure factors were downloaded from the PDB (Berman et al., 2000). The NMR data were taken from reference (Schwalbe et al., 2001). The electrostatic set was excluded because the authors observe that the chemical composition of the sample may be at the basis of the fact that the Pf1 dataset is independent of the others and, therefore, could be not compatible with the others (Higman et al., 2011, Hus et al., 2003), and we did not want to introduce any, even potential, bias. RDCs from mobile residues were excluded according to the analysis reported in references (Bernadó and Blackledge, 2004, Schwalbe et al., 2001). Any manual modification to the structure was performed using COOT (Emsley et al., 2010, Emsley and Cowtan, 2004) and subsequently the structure was re-refined by REFMAC (Murshudov et al., 2011). All joint refinements were performed with REFMAC 5.8.0025.

The agreement to the X-ray data was evaluated in terms of the R-factor (Brunger et al., 1987):

$$R=\frac{\sum_{i}\left|\left|F_{\mathrm{obs},i}\right|-\left|F_{\mathrm{calc},i}\right|\right|}{\sum_{i}\left|F_{\mathrm{obs},i}\right|}$$

where F are the structure factors and the index _i_ runs over the reflections that are used for refinement. Usually these amount to 95% of the total reflections, whereas the remaining randomly selected 5% is used to evaluate the R_free_ (Brünger, 1997).

The agreement between observed (obs) and calculated (calc) RDCs was evaluated in terms of the Q-factor (Cornilescu et al., 1998, Cornilescu and Bax, 2000):

$$Q=\frac{\sqrt{\sum_{i}{\left(obs_{i}-calc_{i}\right)}^{2}}}{\sqrt{\sum_{i}obs_{i}^{2}}}$$

In the REFMAC-NMR implementation published in (Rinaldelli et al., 2014), in order to avoid that introduction of the NMR data could result in the worsening of the geometric parameters, commands were introduced to preserve as much as possible the ideal geometries in placing hydrogen atoms relative to heavier atoms, with two overall weighting parameters over ideal geometries of all atoms involved or not involved in calculation of gradients and second derivatives corresponding to X-ray reflections (WEIGHT REFINED_ATOMS and WEIGHT OTHER_ATOMS, respectively), and three torsion angle restraints, pep1, pep2, and ω to restrain the planarity of the O*_i_*-C*_i_*-N*_i_*_+1_-C*_i_^α^*, of the C*_i_*_-1_-N*_i_*-C*_i_^α^*-H*_i_* and of the C*^α^_i_*-C*_i_*-N*_i_*_+1_-C*^α^_i+1_* groups, respectively. WEIGHT REFINED_ATOMS was set to 1 and WEIGHT OTHER_ATOMS to 100. In the REFMAC refinement, the RDCs were given a weight and a tolerance. The tolerance is set to 0.1 Hz and the weight is adjusted not to cause violation of neither the X-ray term or of the geometry term. Finally, only the direction of the NH bond was refined by the inclusion of the RDCs, and the bond length was set in the calculation of the RDCs to 1.02 Å.

Validation of the structures was performed using MOLPROBITY (Chen et al., 2009).

The crystal contacts were identified through NCONT (Winn et al., 2011).

Hydrogen bonds were evaluated using the DSSP web interface (Joosten et al., 2011, Kabsch and Sander, 1983). According to the description of the web interface, the algorithm discards “any hydrogens present in the input structure and calculates the optimal hydrogen positions by placing them at 1.000 Å from the backbone N in the opposite direction from the backbone C = O bond”.

Back-calculation of RDCs was performed through the FANTEN web interface (Rinaldelli et al., 2015).

Graphs were produced with MatPlotLib (Hunter, 2007), and the rendering of 3D protein structures with UCSF Chimera (Pettersen et al., 2004).

## 3 Results and discussion

The highest resolution structures of hen-egg white lysozyme (HEWL) belong to 4 crystal systems: triclinic, monoclinic, orthorhombic and tetragonal. For each crystal system, we have selected the structure solved at the highest resolution (Table 1).

**Table 1:** PDB entries for HEWL at the highest resolution.

Entry	Crystal system	Resolution/Å	Matthews coefficient	Publication year	Refinement software^a^	Reference
2VB1	Triclinic	0.65	1.69	2007	SHELXL-97 (Sheldrick, 2008)	Wang et al. (2007)
1IEE^b^	Tetragonal	0.94	1.67	2001	SHELXL-97 (Sheldrick, 2008)	Sauter et al. (2001)
3WL2^b^	Monoclinic	0.96	1.79	2014	REFMAC (Kovalevskiy et al., 2018)	N.A.
6F1O	Orthorhombic	0.96	2.2	2018	PHENIX (Liebshner et al., 2019)	Plaza-Garrido et al. (2018)

a All structures were subjected to REFMAC refinement.

b Water molecules present in the deposition were rebuilt.

**Fig. 1:**
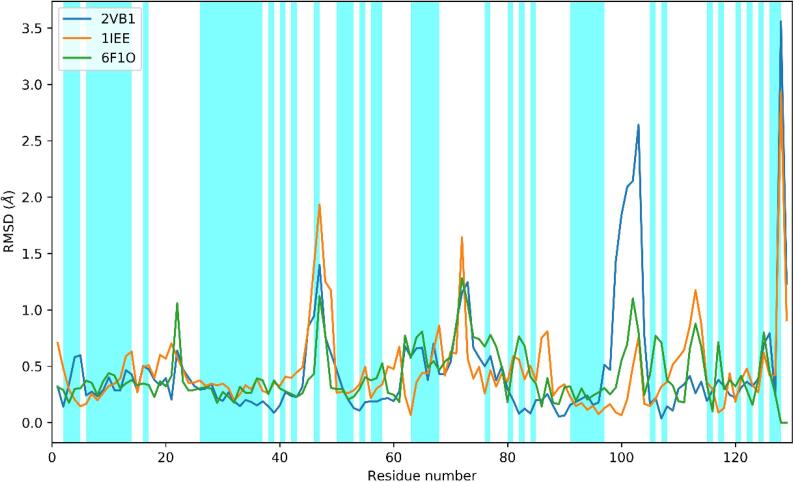
Cα RMSD between each considered structure and 3WL2, which is used as reference (vide infra). Regions for which RDCs are available are shaded in cyan in this figure and colored in green on the structure in Fig. 2. (For interpretation of the references to color in this figure legend, the reader is referred to the web version of this article.)

**Fig. 2:**
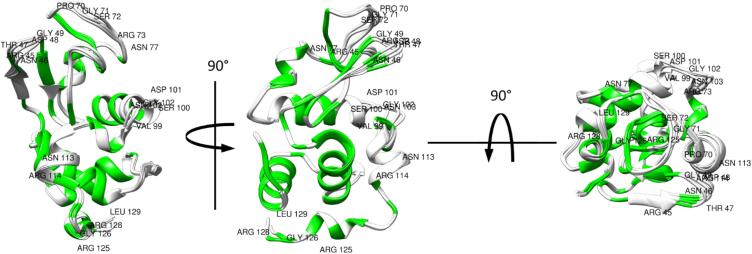
Ribbon representation of the considered lysozyme structures superimposed to 3WL2. Residues in green are those for which RDCs were included in the refinements. Residues with RMSD larger than 1.0 with respect to 3WL2 (see Fig. 1) are labelled. (For interpretation of the references to color in this figure legend, the reader is referred to the web version of this article.)

For checking the agreement of the structures to RDCs prior to refinement, protons, if present in the original file, were removed and repositioned using REDUCE (Word et al., 1999). Then, the structures were re-refined using REFMAC, to minimize the differences due to the refinement software. To minimize the uncertainty in the RDCs that is introduced by mobility, which reduces the value of the RDC with respect to a rigid conformation, we removed the RDCs for the residues indicated by (Bernadó and Blackledge, 2004, Schwalbe et al., 2001). We have also excluded the effect of considering alternate conformations observed in the crystal structures, because we do not want to convey the message that the molecule in solution is the result of averaging between the alternate conformations in the crystal. Even after removing RDCs according to the above criteria, they still encompass regions where a significant RMSD between the different structures is present and they therefore provide enough information for discriminating among the different X-ray structures (Fig. 1, Fig. 2). It is interesting to observe that the agreement (defined as Q-factor) of the different structures to the RDC data is already quite reasonable, in agreement with the prediction by Bax and Zweckstetter (Zweckstetter and Bax, 2002), see Table 2. However, there is no direct correlation between the resolution and the Q-factor, as already highlighted by Schwalbe et al. (Schwalbe et al., 2001).

**Table 2:** Summary of the structure parameters and agreement with the NMR data prior to the refinement with constraints on the geometry of hydrogen placement relative to the backbone atoms.

	2VB1		1IEE		3WL2		6F1O	
	Original	Refined	Original	Refined	Original	Refined	Original	Refined
R_cryst_	0.0848	0.0854	0.1226	0.1252	0.1680	0.1782	0.1376	0.1359
R_free_	0.0843	0.0862	0.1196	0.1321	0.2040	0.1926	0.1474	0.1459
RMS bond length	0.0295	0.0323	0.0156	0.0138	0.0320	0.0173	0.0073	0.0082
RMS bond angle	3.1223	2.9860	2.6764	1.6199	1.9125	1.7180	1.2993	1.352
RMS chiral vol	0.1266	0.1451	0.1210	0.1056	0.1390	0.0827	0.0838	0.0920
Q	0.264	0.263	0.258	0.259	0.246	0.231	0.240	0.241

However, when subjected to joint refinement, the situation changes significantly, and for only two of the four possible structures (3WL2 and 6F1O) the joint refinement improves the agreement to the NMR data without altering the agreement with the X-ray data or with the geometrical parameters (Table 3), whereas 2VB1 cannot be improved and 1IEE is globally worsened with respect to the fit of the X-ray data and of the geometrical parameters. For 2VB1, the decrease of the Q-factor is only marginal and achieved at the price of a sizeable worsening of the agreement to the X-ray data. For 1IEE the situation is more subtle: a reasonably good Q-factor can be achieved, and only the RMS of bond lengths and bond angles is sizably worsened. However, MOLPROBITY validation is impacted dramatically. For the other two structures, the improvement of the agreement to the NMR data is perfectly compatible with the X-ray data as well as with the geometrical parameters, both as evaluated internally in REFMAC and as validated by MOLPROBITY.

**Table 3:** Summary of the structure parameters and agreement with the NMR data after the refinement with constraints on the geometry of hydrogen placement relative to the backbone atoms and after including the NMR data.

	2VB1		1IEE		3WL2^a^		6F1O	
	Refined	Refined with NMR	Refined	Refined with NMR	Refined	Refined with NMR	Refined	Refined with NMR
R_cryst_	0.0854	0.0869	0.1252	0.1216	0.1782	0.1778	0.1359	0.1373
R_free_	0.0862	0.0895	0.1321	0.1327	0.1926	0.1933	0.1459	0.1473
RMS bond length	0.0323	0.0304	0.0138	0.0239	0.0173	0.0198	0.0082	0.0100
RMS bond angle	2.9860	2.7087	1.6199	2.0687	1.7180	1.8920	1.352	1.5273
RMS chiral vol	0.1451	0.1468	0.1056	0.0937	0.0827	0.1046	0.0920	0.1127
Q	0.263	0.187	0.259	0.125	0.231	0.139	0.241	0.075

a NMR restraints were applied to chain A, as it is providing a better agreement (data not shown).

**Fig. 3:**
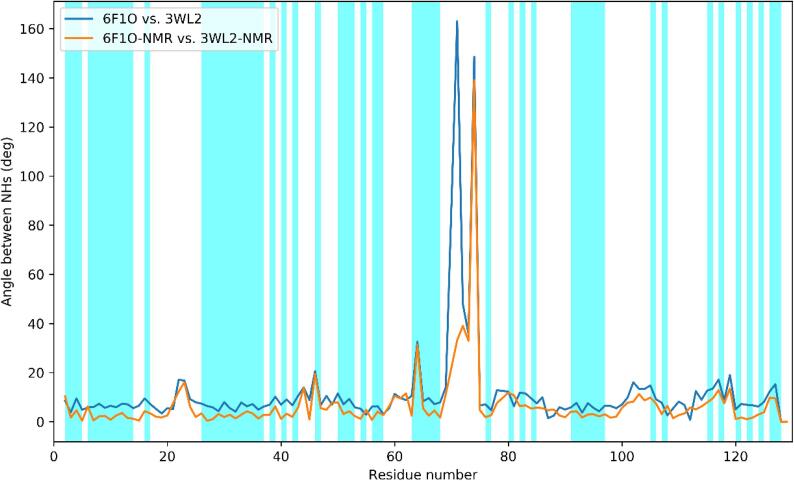
Angles between the NHs between 3WL2 and 6F1O before (blue) and after (orange) the joint refinement. (For interpretation of the references to color in this figure legend, the reader is referred to the web version of this article.)

NMR does not have enough drive to pull the X-ray structure out of the minima defined by the electron density unless large deviations on either the fit to the X-ray data or the ideal geometries are allowed for. The Cα RMSD of the structures before and after the joint refinement amount to 0.016, 0.032, 0.034 and 0.015 A for 2VB1, 1IEE, 3WL2 and 6F1O respectively (the RMSD per residue is shown in Fig. S2) and, even for the residues showing the largest deviations (e.g.: residue 88 for 3WL2) the effect on the electron density map is negligible (see Fig. S3). However, joint refinement can locally bring the structures to convergence, if they can be refined to yield an optimized structure, as it can be seen by comparing the backbone angles and the orientations of the NHs, the differences of which are decreased upon joint refinement (see Supporting Information, Figs. S4–S7). In particular, the difference in NH orientations between 3WL2 and 6F1O is substantially and globally decreased upon joint refinement, as shown in Fig. 3.

**Fig. 4:**
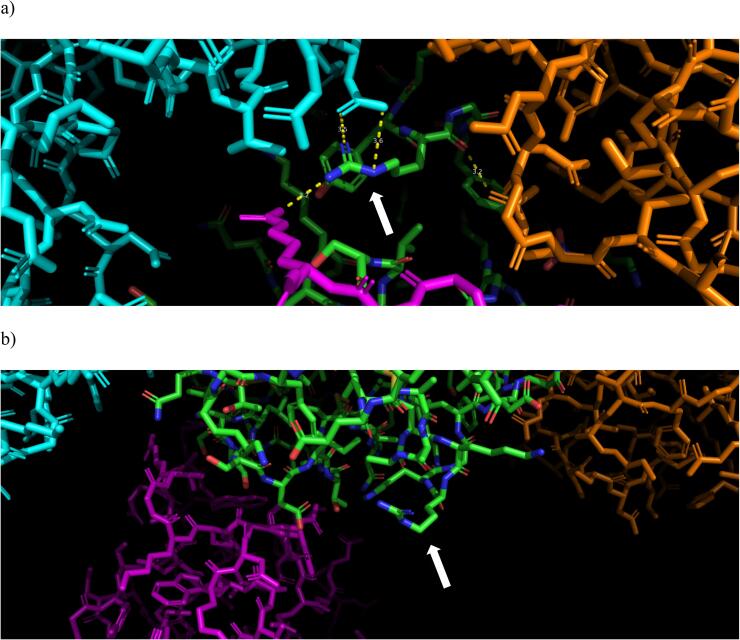
Presence of crystal contacts, indicated with yellow dashes, for residue Arg14 in 2VB1 (panel a) and absence of contacts for the same residue in 6F1O. Arg14 is indicated by an arrow. Crystal symmetry mate molecules are shown in cyan, brown and magenta for both structures. (For interpretation of the references to color in this figure legend, the reader is referred to the web version of this article.)

**Fig. 5:**
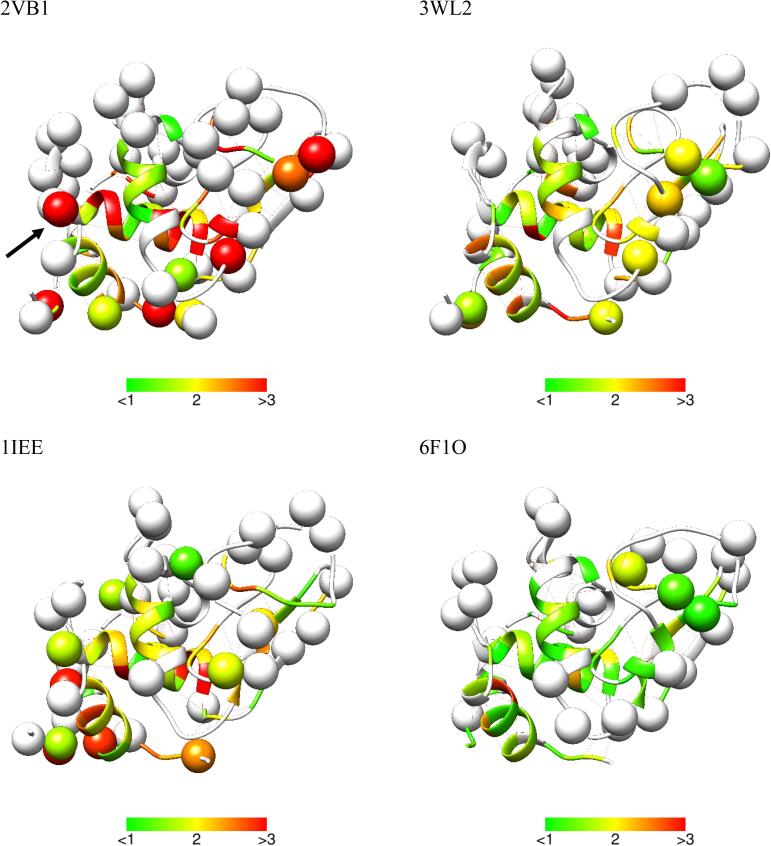
3D structure of lysozyme color coded according to the violation of the RDCs. Residues for which RDCs were not available/used are in white. Residues with > 2 contacts in the crystals are shown as spheres centered on the Cα. It is apparent that the structures with closer packing, where more residues intramolecular contacts, also feature a larger disagreement with the NMR data. Residue Arg14 is marked with a black arrow in the 2VB1 panel (see above, Fig. 4).

It is interesting to observe in this respect that the two structures that yield suboptimal models if subjected to joint refinement (2VB1 and 1IEE) are also the two structures showing the two peaks with the largest deviation in the superposition with respect to 3WL2 (Fig. 1) in the regions 46–52 for 1IEE and 96-105 for 2VB1. A plausible explanation for this could arise from the consideration that the three structures belong to different crystal forms and this in turn implies a different crystal packing. Indeed, 2VB1 and 1IEE have the lower Matthews coefficient (Table 1), therefore a lower solvent content in the crystals. The interactions with symmetry mates in the two above-mentioned regions appears to be more impactful in the case of 2VB1 and 1IEE than with 3WL2 or 6F1O, and these interactions could cause the structure in the crystal to be significantly different from the structure in solution, thus yielding worse joint refinement statistics. As an example, we show the crystal contacts of residue Arg14 in 2VB1 and in 6F1O (Fig. 4, the other contacts are given as supporting information). It is apparent that in 6F1O the crystal contacts that are present in 2VB1 are missing. Even if the RDC for residue 14 is not included in the calculation, the effect of the contacts is apparent in the violation of the RDC of residue 16 in 2VB1 (Fig. 5).

If the residues with a larger number of crystal contacts are plotted against the sequence, together with the deviation of the RDCs, no clear trend emerge (Fig. S8). However, if both data are visualized in the 3D structure of the protein (Fig. 5), the following pattern tends to emerge: residues with crystal contacts (spheres) are either among the residues with large (>1.5 Hz) RDC violations or are connected to them via a hydrogen bonding network.

We performed a structural validation through MOLPROBITY (Chen et al., 2009) (Table 4, see supporting information for the full reports, Tables S1–S6). The MOLPROBITY parameters further confirm that only the 3WL2 and the 6F1O structures satisfy simultaneously the NMR and the X-ray data while preserving, at the same time, a good quality. Overall, MOLPROBITY seems to suggest a slight preference for 3WL2.

**Table 4:** Summary of the Molprobity parameters for the considered structures.

	1IEE			2VB1			3WL2			6F1O		
Parameter	Original	Refined	NMR	Original	Refined	NMR	Original	Refined	NMR	Original	Refined	NMR
target												
**Clashscore percentile**	**35th**	**23rd**	**19th**	**83rd**	**83rd**	**37th**	**0th**	**96th**	**96th**	**98th**	**94th**	**90th**
Poor rotamers	2.10%	1.40%	2.10%	1.32%	0.66%	0%	3.33%	0.48%	0.48%	0%	0%	0%
<0.3%												
Favored rotamers	92.31%	96.50%	95.10%	98.03%	98.03%	98.03%	95.24%	98.57%	98.57%	98.17%	98.17%	99.08%
>98%												
Ramachandran outliers	0%	0%	0%	0.79%	0.79%	0.79%	0%	0.39%	0.39%	0%	0%	0%
<0.05%												
Ramachandran favored	98.43%	98.43%	98.43%	97.64%	98.43%	98.43%	98.43%	98.43%	98.43%	100%	100%	100%
>98%												
**MolProbity score percentile**	**36th**	**43rd**	**31st**	**55th**	**66th**	**31st**	**2nd**	**98th**	**98th**	**99th**	**96th**	**95th**

Finally, we checked the number of hydrogen bonds that can be identified by DSSP analysis and we found that the number of detected hydrogen bonds is changed upon joint refinement (Table 5). Only for 2VB1 the number is decreasing, but this is a reflection of the worsening of the structure.

**Table 5:** Summary of the DSSP report showing the total count of identified hydrogen bonds.

Structure	# hydrogen bonds in the original structure	# hydrogen bonds in the jointly refined structure
2VB1	176	174
1IEE	174	176
3WL2 (two chains)	344	347
6F1O	171	173

Knowing that 3WL2 and 6F1O structures yield improved structures upon joint refinement, we have selected two structures at lower resolution, to verify whether a good agreement could be found as well. For the monoclinic crystal form, we have selected 5LYM (Rao and Sundaralingam, 1996), which has a resolution of 1.8 Å, whereas for the orthorhombic one we have selected the 1WTN (Saijo et al., 2005) structure, which has a deposition resolution of 1.13 Å, but has only 63.9% completeness in the 16.79–1.13 resolution range as detected by EDS.

**Fig. 6:**
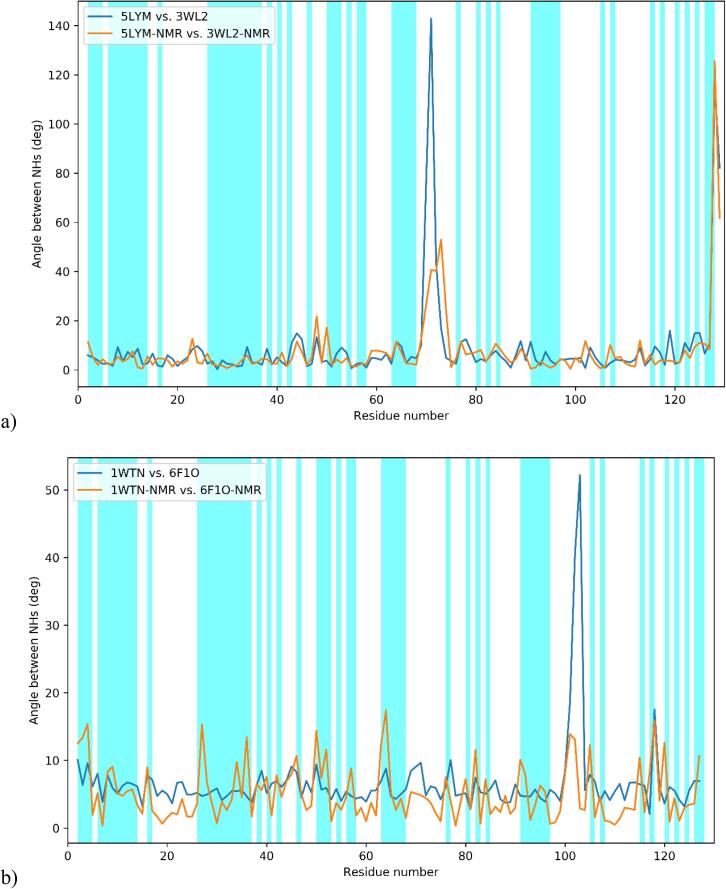
a) Angles between the NHs of 3WL2 and 5LYM before (blue) and after (orange) the joint refinement; b) angles between the NHs of 6F1O and 1WTN before (blue) and after (orange) the joint refinement. (For interpretation of the references to color in this figure legend, the reader is referred to the web version of this article.)

Not surprisingly, both structures are improved by joint refinement. In the case of 5LYM, the clashscore is improved by the inclusion of the NMR data, whereas the MOLPROBITY score is marginally worsened, whereas for 1WTN the clashscore is negligibly worsened but the MOLPROBITY score is greatly improved. Therefore we can conclude that in both cases joint refinement has beneficial results. Again, we can compare the orientation of the NHs before and after the joint refinement, this time comparing the structures belonging to the same crystal form: 5LYM to 3WL2 and 1WTN to 6F1O. The results, shown in Fig. 6, clearly demonstrate that the structures can be brought significantly closer to one another upon inclusion of NMR data.^5^

## 4 Conclusions

This detailed analysis, performed on atomic-resolution X-ray structures, has allowed us to pinpoint that NMR restraints are indeed a valuable contribution not only in the case of medium resolution crystal structures, but also in the case of atomic resolution ones. This implies that it is possible to improve the latter despite it has been generally assumed that their overwhelming amount of data would make crystal structures virtually as accurate as possible on their own.

Also, it appears quite evident from our study, that in the absence of joint refinement all the considered crystal structures appear to agree to the same extent to the NMR data. Thus, NMR data is able to let subtle differences stand out (if any), improving the overall quality of the structure. Finally, for the example of HEWL, we can conclude that crystals forms with larger intermolecular distances and less contacts with symmetry mates, yield lower crystal packing effects and are apparently more representative of the state of the protein in solution.

## CRediT authorship contribution statement

**Antonio Schirò:** Methodology, Software, Investigation, Visualization, Formal analysis. **Azzurra Carlon:** Methodology, Software, Data curation. **Giacomo Parigi:** Conceptualization, Methodology, Supervision. **Garib Murshudov:** Methodology, Software. **Vito Calderone:** Data curation, Formal analysis, Validation, Visualization. **Enrico Ravera:** Conceptualization, Formal analysis, Investigation, Methodology, Supervision, Visualization. **Claudio Luchinat:** Conceptualization, Methodology, Resources, Supervision, Visualization.

## Declaration of Competing Interest

The authors declare that they have no known competing financial interests or personal relationships that could have appeared to influence the work reported in this paper.

## Footnotes

2 Electron microscopy outnumbered the NMR depositions in 2017. In terms of ability of determine the individual atoms positions, it is more similar to X-ray diffraction and we can expect that whatever holds for joint refinement between X-ray and NMR, will hold true for EM and NMR as well.

3 Neutron diffraction can also provide hydrogen positioning (Niimura et al., 2004; Blakeley, 2009; Chen et al., 2012), but the required apparatus is not as widely available as for X-ray and NMR.

4 REFMAC has been used to refine 42.7% of the PDB entries obtained by X-ray.

5 The larger variation in the angles for residues 102–105 for the 1WTN model, that do not appear to cause a significant alteration of the agreement to the X-ray data, can be explained by the very low electron density observed in this area.
