# Supplementary material for: On the complementarity of X-ray and NMR data
Source: J Struct Biol X. 2020 Jan 7;4:100019. doi: 10.1016/j.yjsbx.2020.100019 (PMC7337059; doi:10.1016/j.yjsbx.2020.100019)
Supplement: Supplementary data 1 [file mmc1.docx]

| 2VB1 | 3WL2 |
| --- | --- |
| 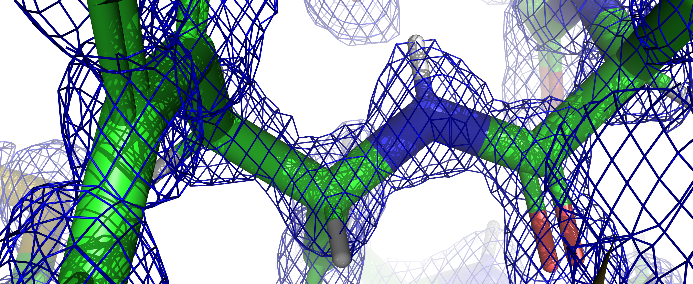 | 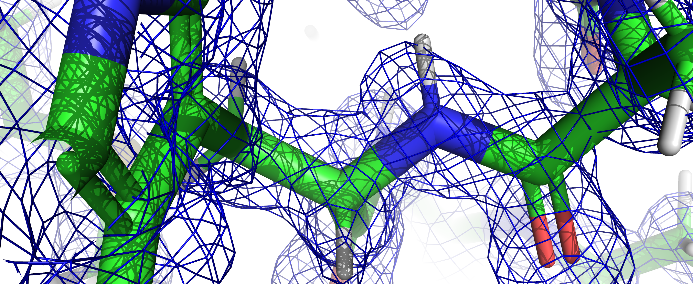 |
| 6F1O | 1IEE |
| 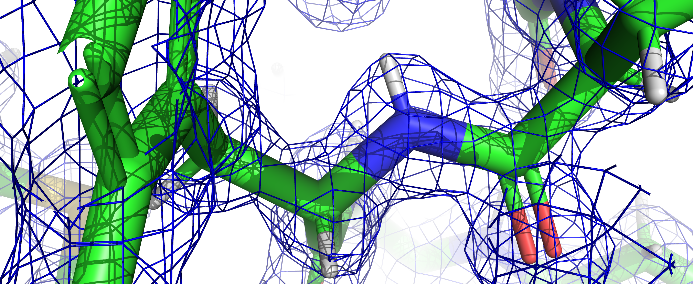 | 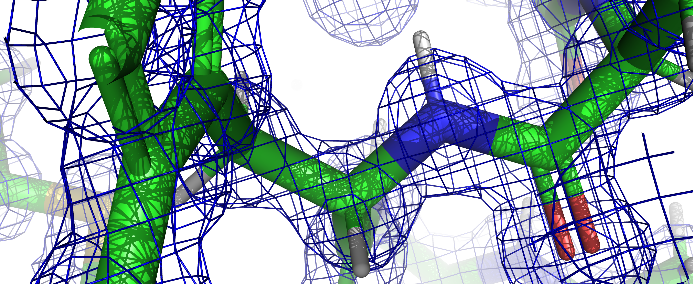 |

Figure S1. Electron density (2F_O_-F_C_ contoured at 1σ) around the backbone atoms for a residue (W28, which has a thermal factor of 3 in the structure 2VB1) for all the four considered high-resolution structures. Clearly the hydrogen positioning cannot be recovered on the sole basis of the electron density.


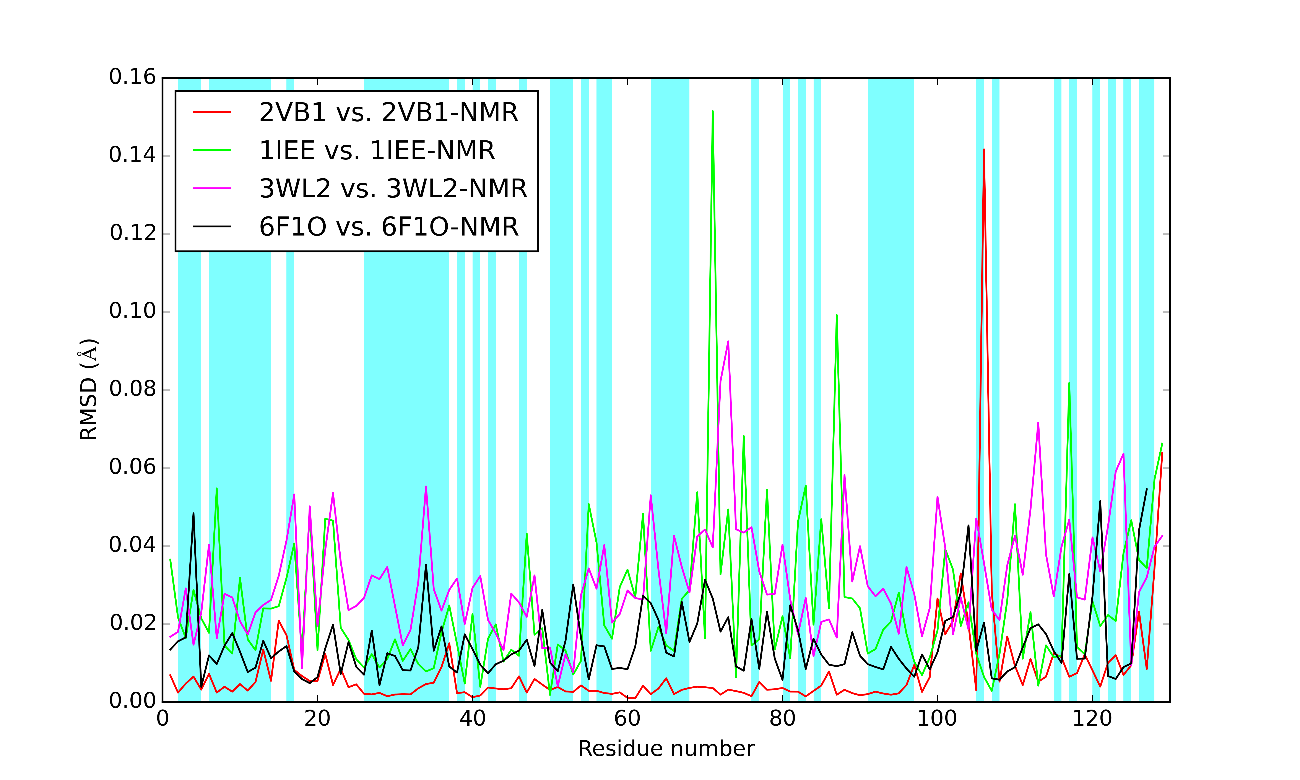


Figure S2. RMSD per residue for the selected structures between the model obtained by X-ray only and the model obtained by joint refinement.

| Before joint refinement | After joint refinement |
| --- | --- |
| 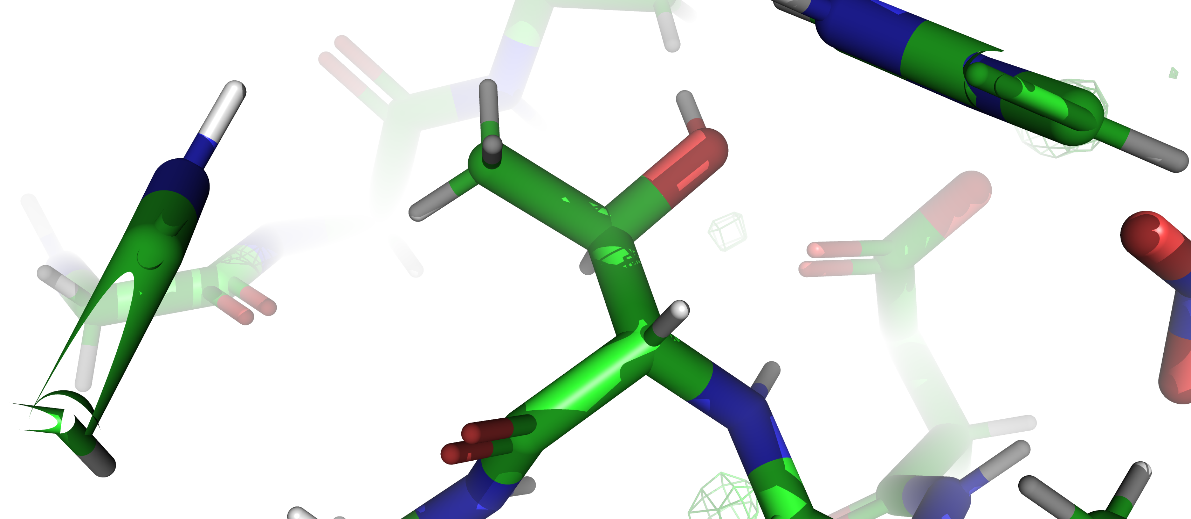 | 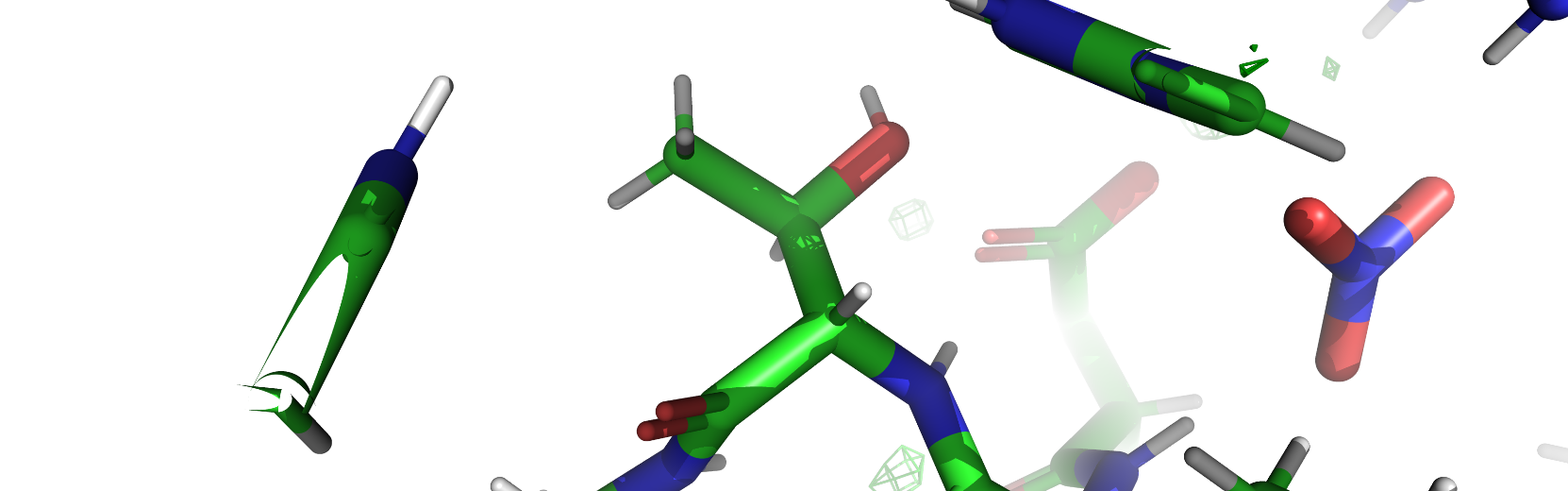 |
| 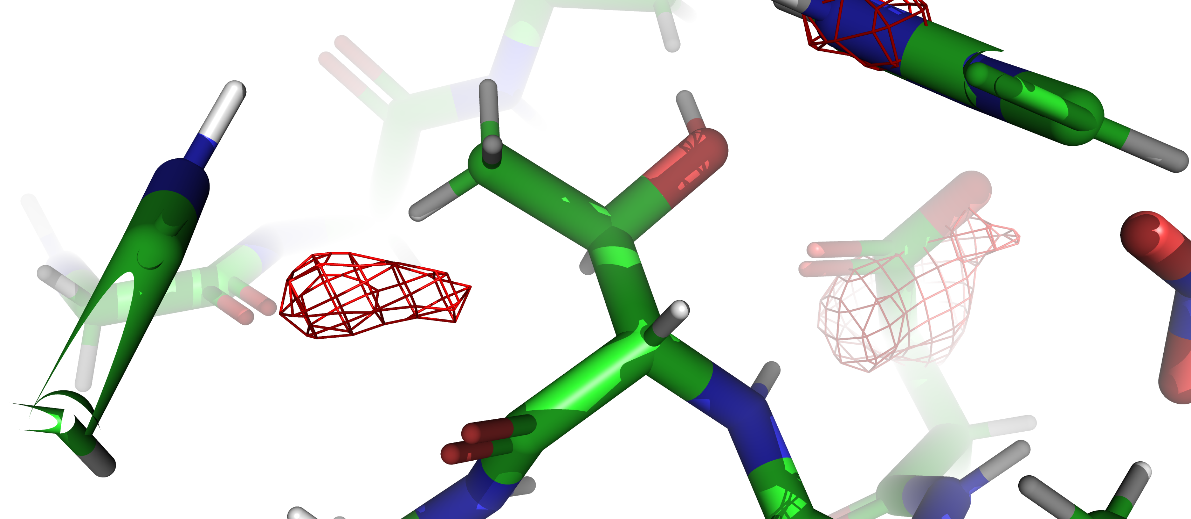 | 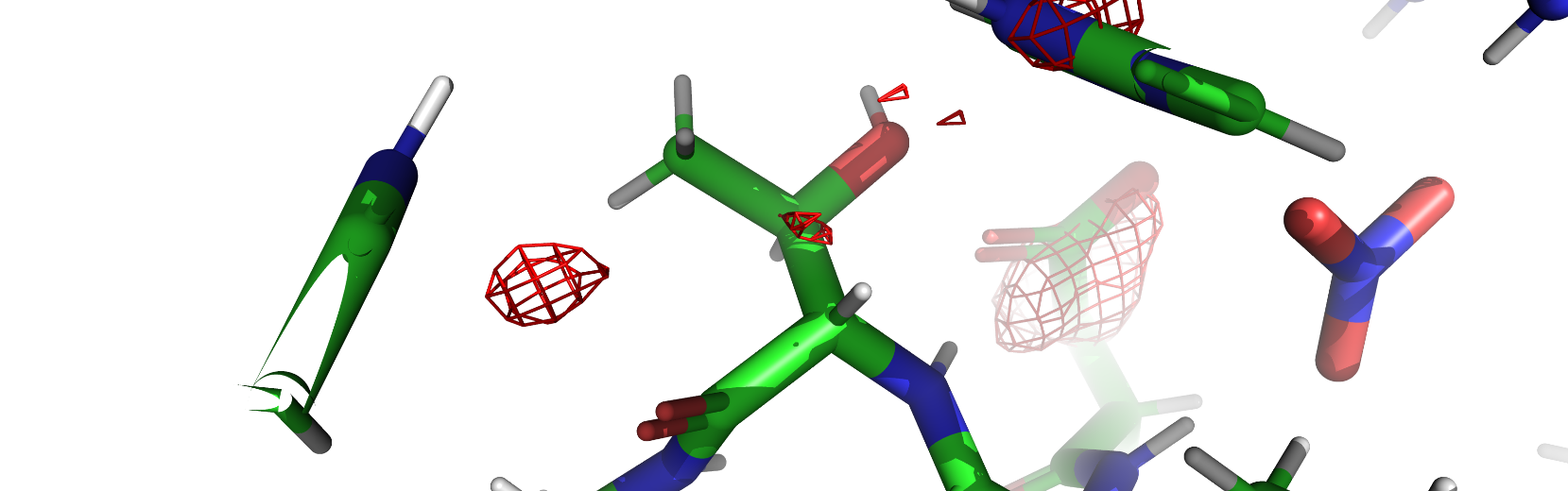 |

Figure S3. Difference electron density map (F_O_-F_C_ contoured at ±3σ – top, positive contour in green; bottom – negative contour in red) around the atoms of residue 88 in 3WL2 before and after joint refinement.
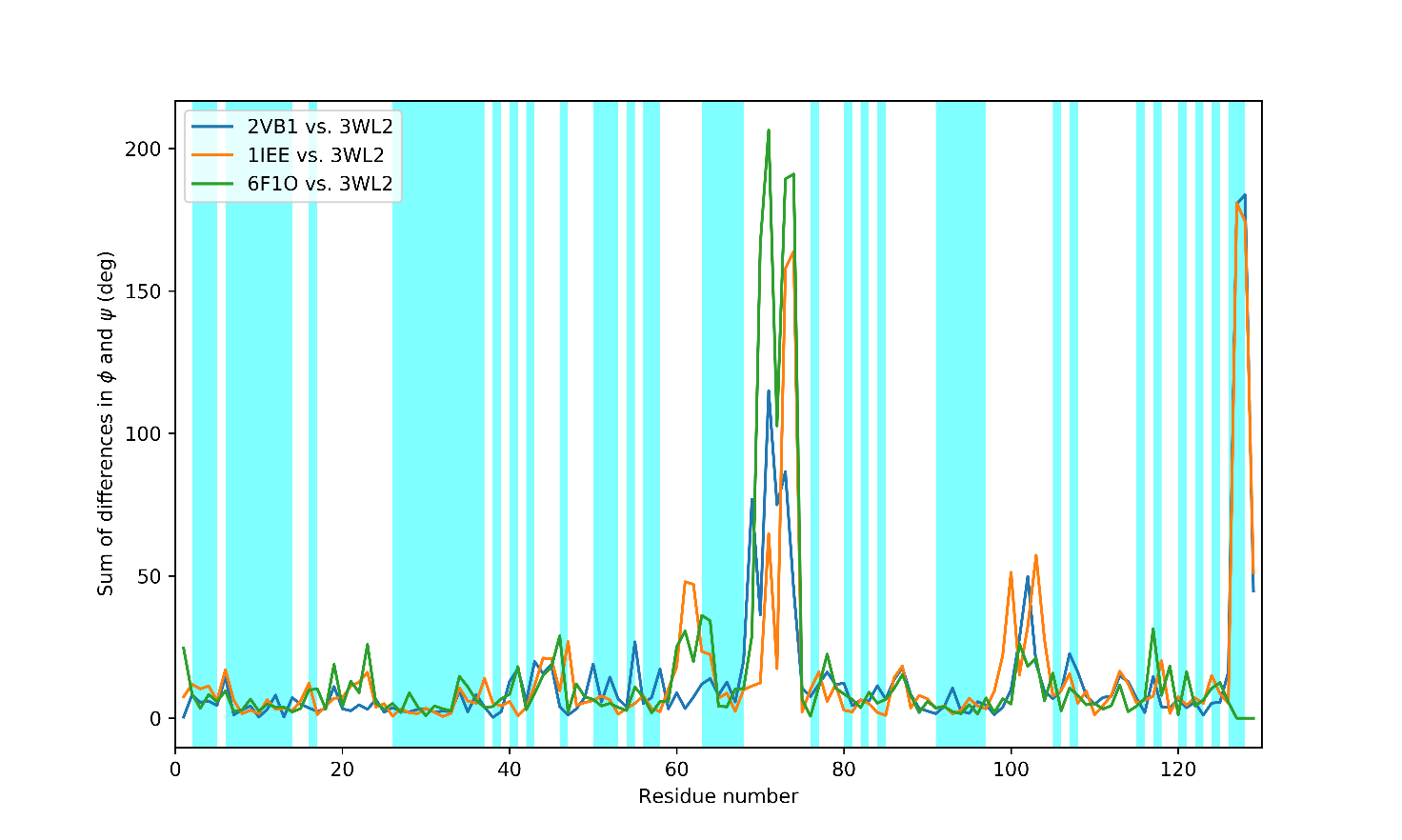


Figure S4. Sum of the absolute values of the differences in φ and ψ angles along the different residues for the deposited 2VB1, 1IEE and 6F1O structures with respect to the deposited 3WL2.


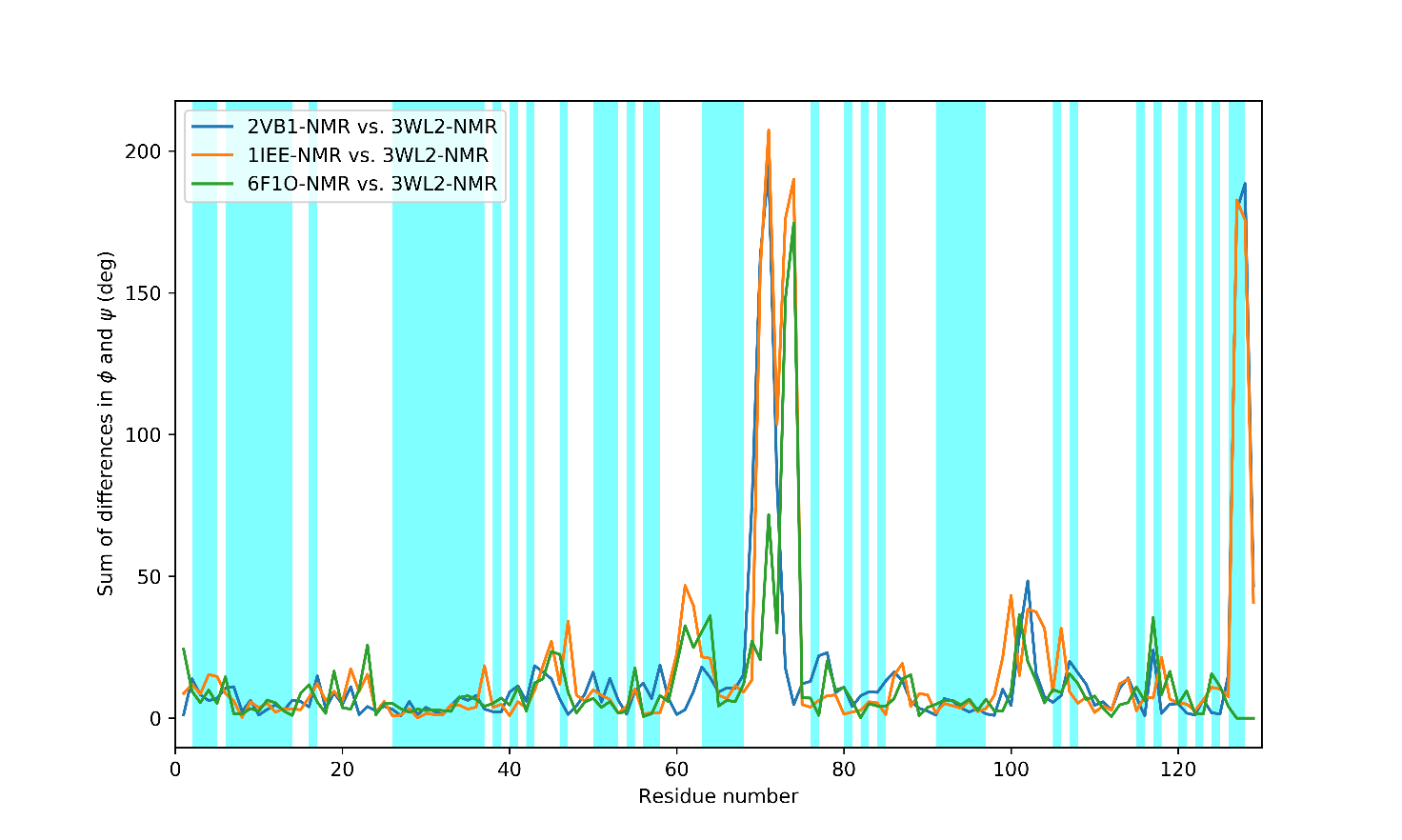


Figure S5. Sum of the absolute values of the differences in φ and ψ angles along the different residues for the 2VB1, 1IEE and 6F1O structures with respect to the 3WL2 structure after joint refinement. Color code is same as Figure S4. Of note, the difference for 6F1O (green line) is markedly reduced.


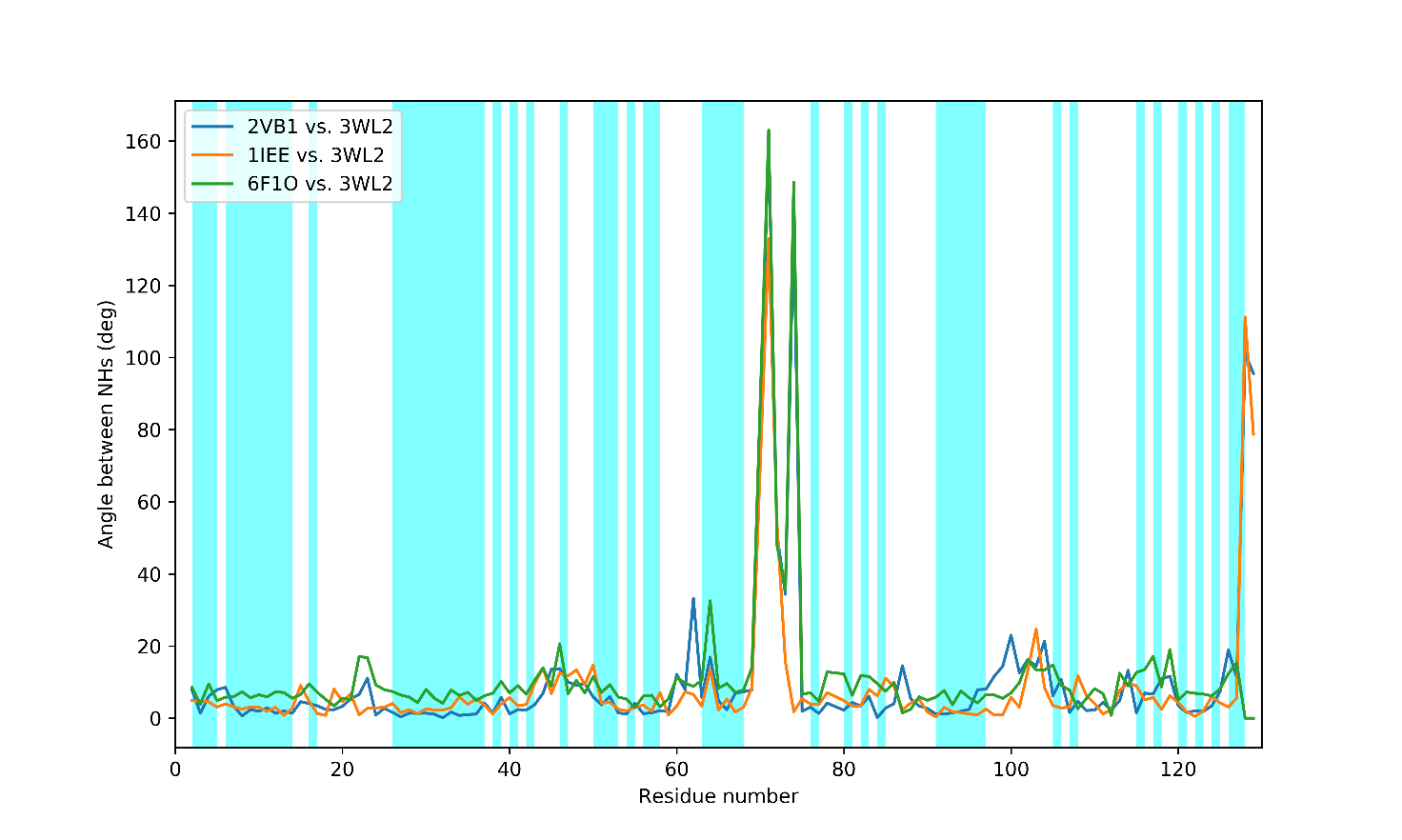


Figure S6. Angle between the NHs of the deposited 2VB1, 1IEE and 6F1O structures with respect to the deposited 3WL2 used as reference. The green line is reported in figure 3 in the main text.


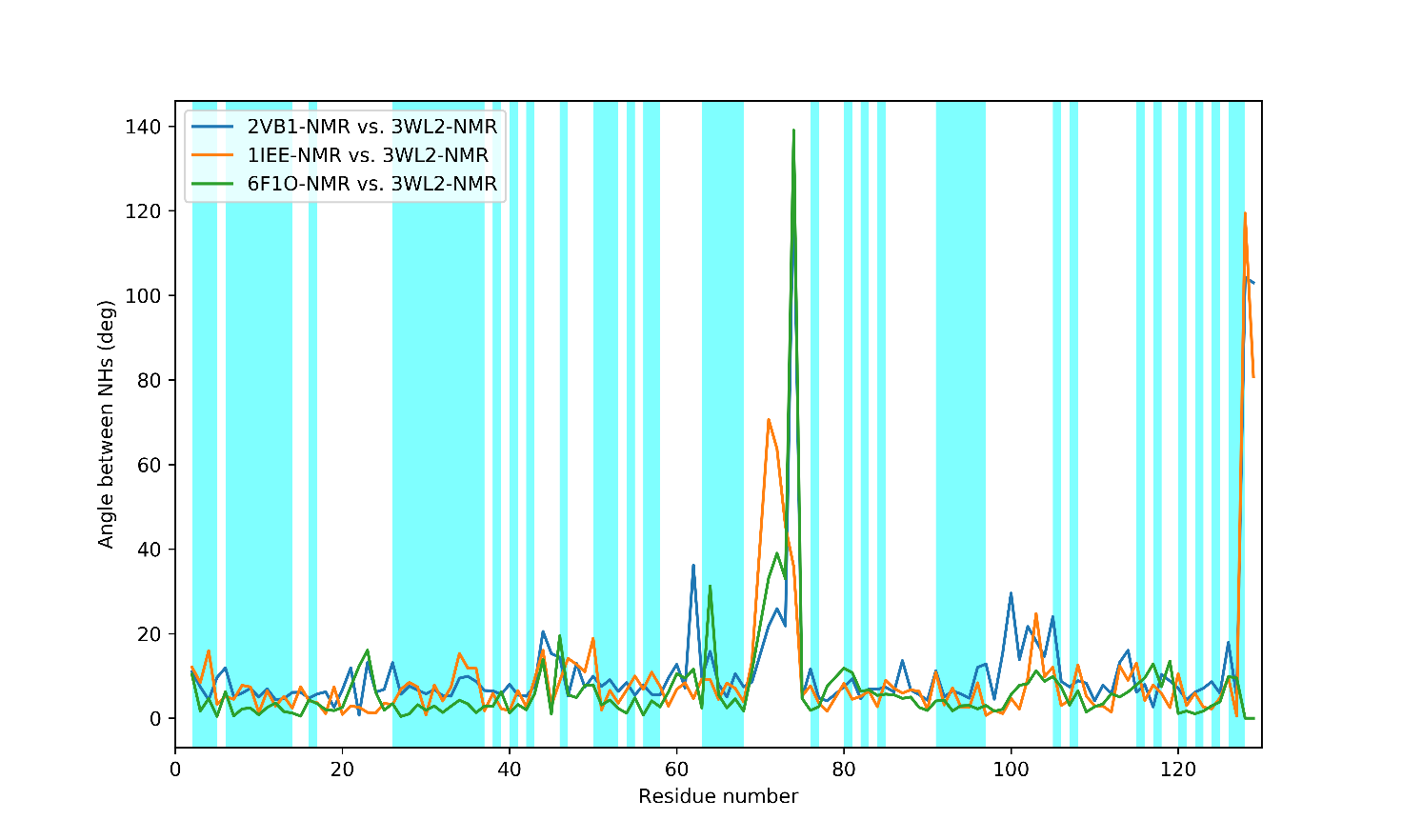


Figure S7. Angle between the NHs of the 2VB1, 1IEE and 6F1O structures with respect to the 3WL2 used as reference, after joint refinement. Color code is same as Figure S6. The green line is reported in figure 3 in the main text.

| a) 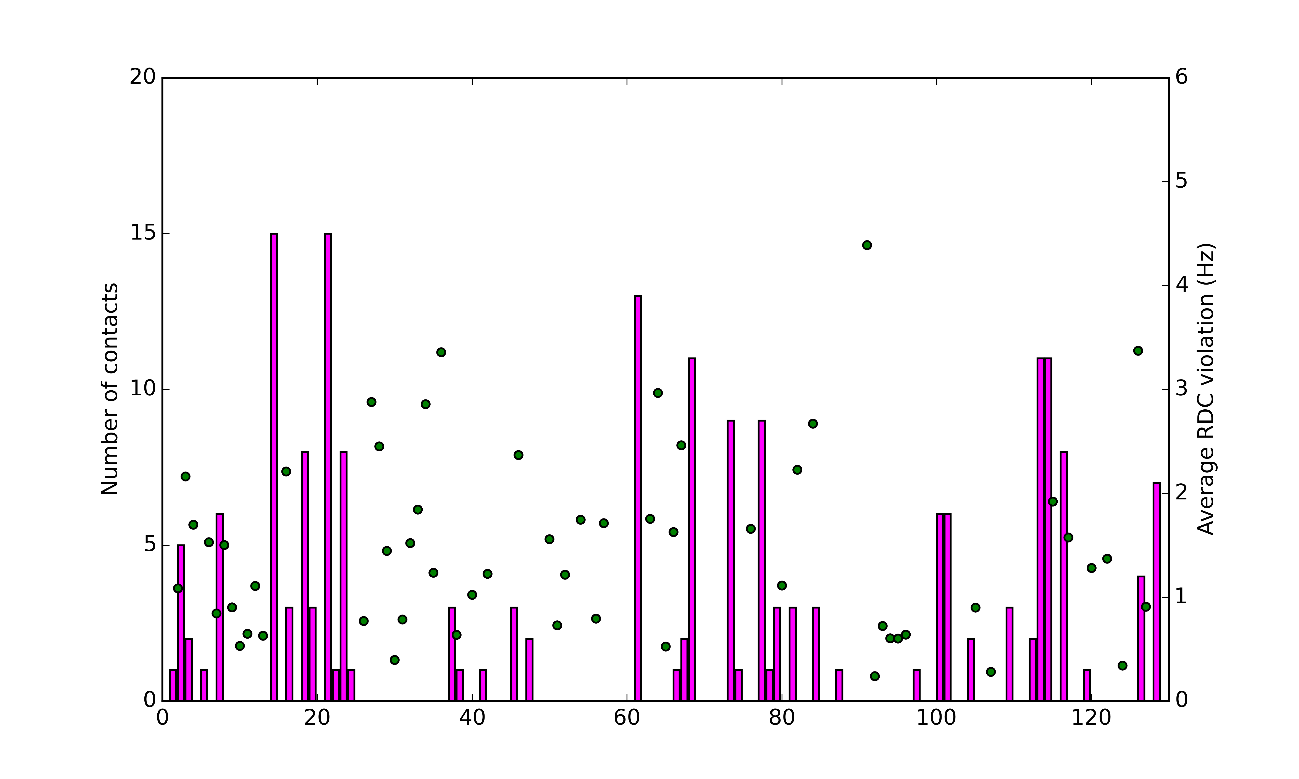 |
| --- |
| b) 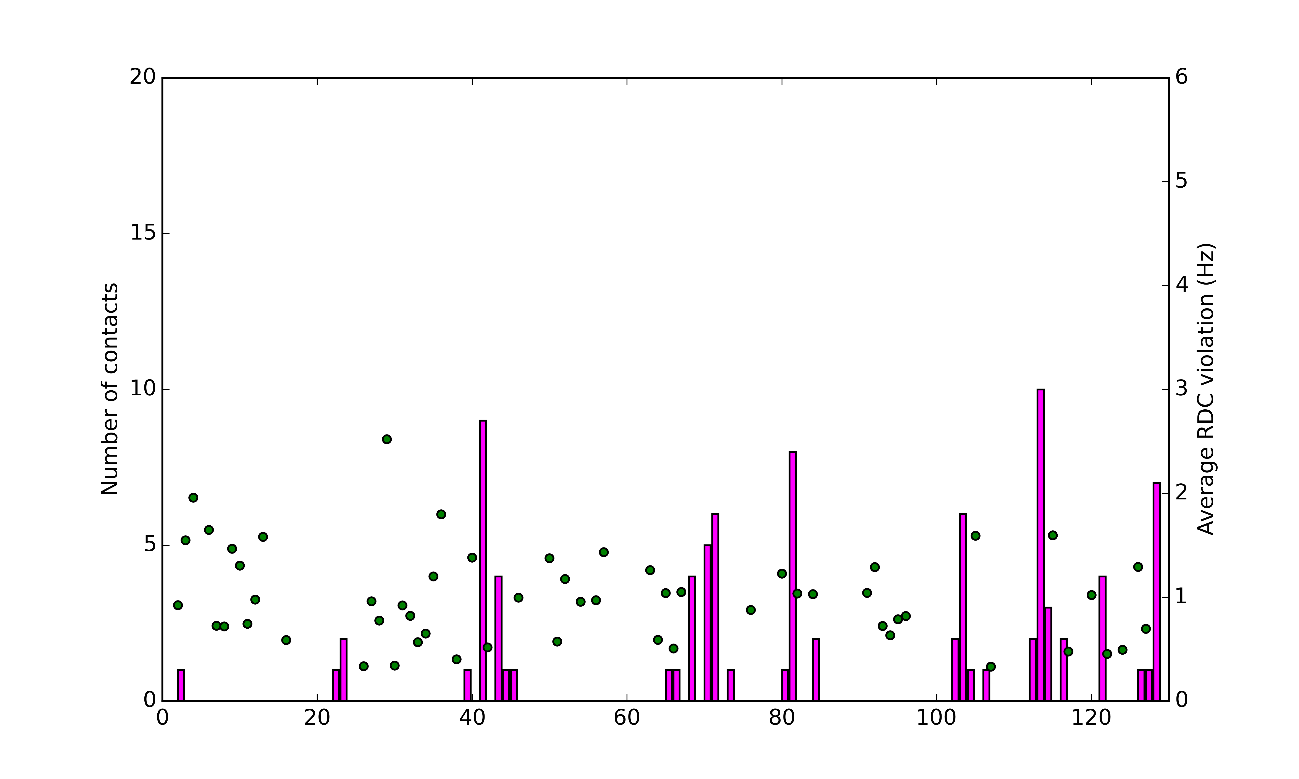 |
| c) 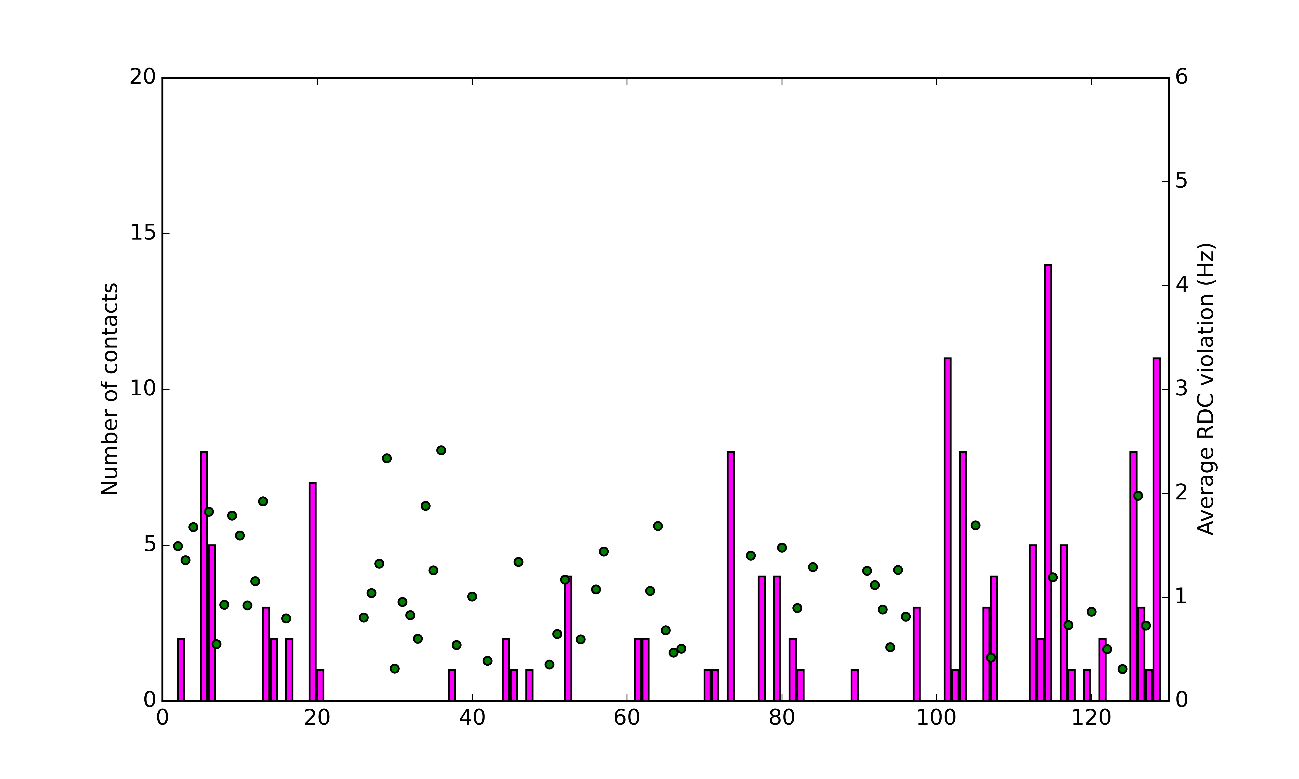 |
| d) 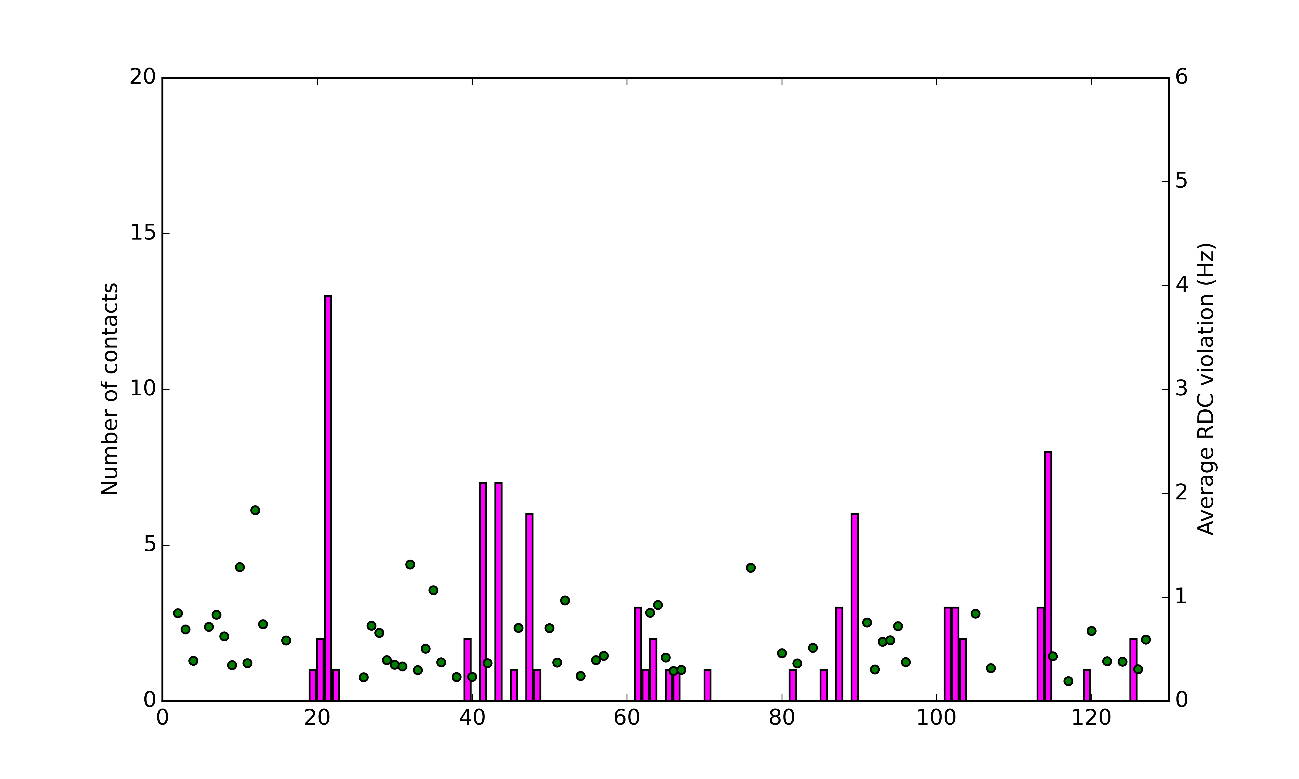 |

Figure S8. Number of contacts (bars) and RDC violation (dots, average over the 7 datasets) after joint refinement for each residue in the HEWL sequence (a) 2VB1, (b) 1IEE, (c) 3WL2, (d) 6F1O.

Table S1. Molprobity report for 1IEE

|  | | PDB code: 1IEE – Resolution: 0.94 Å | | | | | | |
| --- | --- | --- | --- | --- | --- | --- | --- | --- |
| Parameters | Goal | | PDB Untouched | | REFMAC 5.8.0025 refinement  Reduce applied | | REFMAC 5.8.0025 refinement +NMR  Reduce applied | |
| **Clashscore, all atoms** |  | | **7.73** | **35th percentile* (N=126, 0.94Å ± 0.25Å)** | **9.35** | **23rd percentile (N=126, 0.94Å ± 0.25Å)** | **10.98** | **19th percentile (N=126, 0.94Å ± 0.25Å)** |
| Poor rotamers | <0.3% | | 3 | 2.10% | 2 | 1.40% | 3 | 2.10% |
| Favored rotamers | >98% | | 132 | 92.31% | 138 | 96.50% | 136 | 95.10% |
| Ramachandran outliers | <0.05% | | 0 | 0% | 0 | 0% | 0 | 0% |
| Ramachandran favored | >98% | | 125 | 98.43% | 125 | 98.43% | 125 | 98.43% |
| **MolProbity score** |  | | **1.74** | **36th percentile* (N=449, 0.94Å ± 0.25Å)** | **1.68** | **43rd percentile (N=449, 0.94Å ± 0.25Å)** | **1.80** | **31st percentile (N=449, 0.94Å ± 0.25Å)** |
| Cβ deviations > 0.25 Å | Goal: 0 | | 3 | 1.99% | 1 | 0.66% | 1 | 0.66% |
| Bad bonds | Goal: 0% | | 2/1264 | 0.16% | 5/1264 | 0.40% | 13/1264 | 1.03% |
| Bad angles | Goal: <0.1% | | 33/1727 | 1.91% | 3/1727 | 0.17% | 12/1727 | 0.69% |
| Cis Prolines | Expected: ≤1 per chain, or ≤5% | | 0/3 | 0% | 0/3 | 0% | 0/3 | 0% |
| R_free_/R_cryst_ |  | | 0.151/0.123 | | 0.132/0.125 | | 0.132/0.121 | |
| RMSD bond length (Å) |  | | 0.016 | | 0.014 | | 0.024 | |
| RMSD bond angles (°) |  | | --- | | 1.620 | | 2.069 | |

Table S2. Molprobity report for 2VB1

|  | | PDB code: 2VB1 – Resolution: 0.65 Å | | | | | | |
| --- | --- | --- | --- | --- | --- | --- | --- | --- |
| Parameters | Goal | | PDB Untouched | | REFMAC 5.8.0025 refinement  Reduce applied | | REFMAC 5.8.0025 refinement +NMR  Reduce applied | |
| **Clashscore, all atoms** |  | | **3.26** | **83rd percentile* (N=24, 0Å - 0.9Å)** | **3.63** | **83rd percentile* (N=24, 0Å - 0.9Å)** | **7.98** | **37th percentile* (N=24, 0Å - 0.9Å)** |
| Poor rotamers | <0.3% | | 2 | 1.32% | 1 | 0.66% | 0 | 0% |
| Favored rotamers | >98% | | 149 | 98.03% | 149 | 98.03% | 149 | 98.03% |
| Ramachandran outliers | <0.05% | | 1 | 0.79% | 1 | 0.79% | 1 | 0.79% |
| Ramachandran favored | >98% | | 124 | 97.64% | 125 | 98.43% | 125 | 98.43% |
| **MolProbity score** |  | | **1.60** | **55th percentile* (N=158, 0.4Å - 1Å)** | **1.49** | **66th percentile* (N=158, 0.4Å - 1Å)** | **1.77** | **31st percentile* (N=158, 0.4Å - 1Å)** |
| Cβ deviations > 0.25 Å | Goal: 0 | | 8 | 4.94% | 9 | 5.56% | 11 | 6.79% |
| Bad bonds | Goal: 0% | | 24/1418 | 1.69% | 33/1418 | 2.33% | 34/1418 | 2.40% |
| Bad angles | Goal: <0.1% | | 65/1938 | 3.35% | 68/1938 | 3.51% | 47/1938 | 2.43% |
| Cis Prolines | Expected: ≤1 per chain, or ≤5% | | 0/3 | 0% | 0/3 | 0% | 0/3 | 0% |
| R_free_/R_cryst_ |  | | 0.095/0.084 | | 0.086/0.085 | | 0.089/0.087 | |
| RMSD bond length (Å) |  | | 0.024 | | 0.032 | | 0.030 | |
| RMSD bond angles (°) |  | | --- | | 2.986 | | 2.709 | |

Table S3. Molprobity report for 3WL2

|  | | PDB code: 3WL2 – Resolution: 0.96 Å | | | | | | |
| --- | --- | --- | --- | --- | --- | --- | --- | --- |
| Parameters | Goal | | PDB Untouched | | REFMAC 5.8.0025 refinement  Reduce applied | | REFMAC 5.8.0025 refinement +NMR  Reduce applied | |
| **Clashscore, all atoms** |  | | **48.87** | **0th percentile* (N=157, 0.96Å ± 0.25Å)** | **1.51** | **96th percentile* (N=157, 0.96Å ± 0.25Å)** | **1,76** | **96th percentile* (N=157, 0.96Å ± 0.25Å)** |
| Poor rotamers | <0.3% | | 7 | 3.33% | 1 | 0.48% | 1 | 0.48% |
| Favored rotamers | >98% | | 200 | 95.24% | 207 | 98.57% | 207 | 98.57% |
| Ramachandran outliers | <0.05% | | 0 | 0% | 1 | 0.39% | 1 | 0.39% |
| Ramachandran favored | >98% | | 250 | 98.43% | 250 | 98.43% | 250 | 98.43% |
| **MolProbity score** |  | | **2.56** | **2nd percentile* (N=584, 0.96Å ± 0.25Å)** | **0.89** | **98th percentile* (N=584, 0.96Å ± 0.25Å)** | **0.93** | **98th percentile* (N=584, 0.96Å ± 0.25Å)** |
| Cβ deviations > 0.25 Å | Goal: 0 | | 2 | 0.85% | 0 | 0% | 0 | 0% |
| Bad bonds | Goal: 0% | | 20/2081 | 0.96% | 20/2081 | 0.96% | 20/2081 | 0.96% |
| Bad angles | Goal: <0.1% | | 6/2806 | 0.21% | 4/2806 | 0.14% | 4/2806 | 0.14% |
| Cis Prolines | Expected: ≤1 per chain, or ≤5% | | 0/4 | 0% | 0/4 | 0% | 0/4 | 0% |
| R_free_/R_cryst_ |  | | 0.195/0.160 | | 0.192/0.178 | | 0.193/0.178 | |
| RMSD bond length (Å) |  | | 0.021 | | 0.017 | | 0.020 | |
| RMSD bond angles (°) |  | | 1.914 | | 1.717 | | 1.892 | |

Table S4. Molprobity report for 6F1O

|  | | PDB code: 6F1O – Resolution: 0.96 Å | | | | | | |
| --- | --- | --- | --- | --- | --- | --- | --- | --- |
| Parameters | Goal | | PDB Untouched | | REFMAC 5.8.0025 refinement  Reduce applied | | REFMAC 5.8.0025 refinement +NMR  Reduce applied | |
| **Clashscore, all atoms** |  | | **0.99** | **98th percentile* (N=157, 0.96Å ± 0.25Å)** | **2.47** | **94th percentile* (N=157, 0.96Å ± 0.25Å)** | **2.96** | **90th percentile* (N=157, 0.96Å ± 0.25Å)** |
| Poor rotamers | <0.3% | | 0 | 0% | 0 | 0% | 0 | 0% |
| Favored rotamers | >98% | | 107 | 98.17% | 107 | 98.17% | 108 | 99.08% |
| Ramachandran outliers | <0.05% | | 0 | 0% | 0 | 0% | 0 | 0% |
| Ramachandran favored | >98% | | 125 | 100% | 125 | 100% | 125 | 100% |
| **MolProbity score** |  | | **0.79** | **99th percentile* (N=584, 0.96Å ± 0.25Å)** | **1.03** | **96th percentile* (N=584, 0.96Å ± 0.25Å)** | **1.09** | **95th percentile* (N=584, 0.96Å ± 0.25Å)** |
| Cβ deviations > 0.25 Å | Goal: 0 | | 0 | 0% | 0 | 0% | 1 | 0.83% |
| Bad bonds | Goal: 0% | | 0/1064 | 0% | 0/1064 | 0% | 0/1064 | 0.77% |
| Bad angles | Goal: <0.1% | | 0/1451 | 0% | 0/1451 | 0% | 0/1451 | 0.72% |
| Cis Prolines | Expected: ≤1 per chain, or ≤5% | | 0/2 | 0% | 0/2 | 0% | 0/2 | 0% |
| R_free_/R_cryst_ |  | | 0.142/0.132 | | 0.145/0.135 | | 0.146/0.135 | |
| RMSD bond length (Å) |  | | --- | | 0.008 | | 0.014 | |
| RMSD bond angles (°) |  | | --- | | 1.352 | | 1.634 | |

Table S5. Molprobity report for 5LYM

|  | | PDB code: 5LYM – Resolution: 1.80 Å | | | | | | |
| --- | --- | --- | --- | --- | --- | --- | --- | --- |
| Parameters | Goal | | PDB Untouched | | REFMAC 5.8.0025 refinement  Reduce applied | | REFMAC 5.8.0025 refinement +NMR  Reduce applied | |
| **Clashscore, all atoms** |  | | **7.61** | **87th percentile* (N=837, 1.80Å ± 0.25Å)** | **7.86** | **86th percentile* (N=839, 1.78Å ± 0.25Å)** | **6.84** | **90th percentile* (N=839, 1.78Å ± 0.25Å)** |
| Poor rotamers | <0.3% | | 18 | 8.57% | 18 | 8.57% | 18 | 8.75% |
| Favored rotamers | >98% | | 182 | 86.67% | 177 | 84.29% | 177 | 84.29% |
| Ramachandran outliers | <0.05% | | 1 | 0.39% | 0 | 0% | 0 | 0% |
| Ramachandran favored | >98% | | 249 | 98.03% | 249 | 98.03% | 247 | 97.24% |
| **MolProbity score** |  | | **2.13** | **52nd percentile* (N=11444, 1.80Å ± 0.25Å)** | **2.14** | **51st percentile* (N=11266, 1.78Å ± 0.25Å)** | **2.23** | **43rd percentile* (N=11266, 1.78Å ± 0.25Å)** |
| Cβ deviations > 0.25 Å | Goal: 0 | | 8 | 3.42% | 6 | 2.56% | 5 | 2.14% |
| Bad bonds | Goal: 0% | | 23/2068 | 1.11% | 14/2068 | 0.68% | 16/2068 | 0.77% |
| Bad angles | Goal: <0.1% | | 82/2796 | 2.93% | 16/2796 | 0.57% | 20/2796 | 0.72% |
| Cis Prolines | Expected: ≤1 per chain, or ≤5% | | 0/4 | 0% | 0/4 | 0% | 0/4 | 0% |
| R_free_/R_cryst_ |  | | 0.264/0.189 | | 0.211/0.155 | | 0.211/0.155 | |
| RMSD bond length (Å) |  | | 0.013 | | 0.020 | | 0.021 | |
| RMSD bond angles (°) |  | | 2.800 | | 2.309 | | 2.383 | |

Table S6. Molprobity report for 1WTN

|  | | PDB code: 1WTN – Resolution: 1.13 Å | | | | | | |
| --- | --- | --- | --- | --- | --- | --- | --- | --- |
| Parameters | Goal | | PDB Untouched | | REFMAC 5.8.0025 refinement  Reduce applied | | REFMAC 5.8.0025 refinement +NMR  Reduce applied | |
| **Clashscore, all atoms** |  | | **4.52** | **79th percentile* (N=224, 1.13Å ± 0.25Å)** | **3.01** | **92nd percentile* (N=224, 1.13Å ± 0.25Å)** | **3.02** | **92nd percentile* (N=224, 1.13Å ± 0.25Å)** |
| Poor rotamers | <0.3% | | 1 | 0.92% | 3 | 2.75% | 1 | 0.92% |
| Favored rotamers | >98% | | 106 | 97.25% | 104 | 95.41% | 104 | 95.41% |
| Ramachandran outliers | <0.05% | | 1 | 0.79% | 0 | 0% | 0 | 0% |
| Ramachandran favored | >98% | | 125 | 98.43% | 127 | 100% | 127 | 100% |
| **MolProbity score** |  | | **1.23** | **91st percentile* (N=976, 1.13Å ± 0.25Å)** | **1.43** | **76th percentile* (N=976, 1.13Å ± 0.25Å)** | **1.09** | **96th percentile* (N=976, 1.13Å ± 0.25Å)** |
| Cβ deviations > 0.25 Å | Goal: 0 | | 0 | 0% | 2 | 1.71% | 1 | 0.85% |
| Bad bonds | Goal: 0% | | 0/1033 | 0% | 0/1033 | 0% | 0/1033 | 0% |
| Bad angles | Goal: <0.1% | | 6/1400 | 0.43% | 6/1400 | 0.43% | 4/1400 | 0.29% |
| Cis Prolines | Expected: ≤1 per chain, or ≤5% | | 0/2 | 0% | 0/2 | 0% | 0/2 | 0% |
| R_free_/R_cryst_ |  | | 0.200/0.170 | | 0.168/0.125 | | 0.168/0.125 | |
| RMSD bond length (Å) |  | | 0.005 | | 0.010 | | 0.011 | |
| RMSD bond angles (°) |  | | 1.818 | | 1.566 | | 1.571 | |
